# Brain volume loss in Japanese patients with multiple sclerosis is present in the early to middle stage of the disease

**DOI:** 10.1016/j.heliyon.2024.e28136

**Published:** 2024-03-18

**Authors:** Juichi Fujimori, Ichiro Nakashima

**Affiliations:** Division of Neurology, Tohoku Medical and Pharmaceutical University, Sendai, Japan

**Keywords:** Multiple sclerosis, MRI, Volumetric analysis, Ico**brain ms**, cluster analysis

## Abstract

**Background:**

To determine which disease-modifying therapies should be used in patients with multiple sclerosis (MS), identifying patients at high and low risk for brain volume loss (BVL) is important. Although the BVL rate in MS is nearly constant from early to late disease onset, regardless of the disease stage, individual differences have been noted. Moreover, as disease duration increases, the risk of chronic progression increases, and brain atrophy becomes more noticeable. Therefore, measuring prognosis using a classification that considers BVL rate and disease duration is appropriate. We aimed to investigate the BVL in Japanese patients with MS.

**Methods:**

Herein, with an observational period of approximately 3.5 years, 82 Japanese patients with MS were included. The volumes and annualised volume changes (AVCs) of the grey matter (GM) and whole brain were evaluated using ico**brain ms.**

**Results:**

Whole-brain AVCs varied, especially among patients with a disease duration within approximately 16 years. Cluster analysis using two variables, disease duration and whole-brain AVC, identified the SM (short to middle duration and mild atrophy rates), SS (short to middle duration and severe atrophy rates), and L (long duration) groups. The optimal cut-off values for disease duration and whole-brain AVC to discriminate among the three groups were 15.8 years and −0.43%, respectively. Compared with the SM group, the SS group had higher Multiple Sclerosis Severity Scale (MSSS) and Expanded Disability Status Scale (EDSS) scores, lower information processing speed (IPS), higher lesion loads, higher whole-brain and GM volume loss, and higher GM atrophy rates. Moreover, among the 63 patients with MS included in the SM and SS groups, whole-brain AVCs were significantly correlated with the EDSS and MSSS scores and IPS.

**Conclusion:**

BVL rates vary, especially among Japanese patients with MS with short to middle disease duration, and BVL degree is associated with poor prognosis.

## Highlights

• Identifying patients with MS at risk for brain volume loss (BVL) is important.
• Ico**brain ms** was used to examine volumes and AVCs of grey matter and whole brain.
• Whole-brain AVCs varied among patients with a disease duration within 16 years.
• BVL rates vary among Japanese patients with MS with short disease duration.
• The degree of BVL is associated with poor prognosis.

## 1 Introduction

Multiple sclerosis (MS) is a chronic inflammatory, demyelinating, and neurodegenerative disease of the central nervous system [1]. Neurodegenerative changes, such as brain atrophy in several regions, including the deep grey matter, can already be observed in the early stages of MS [2]. Brain atrophy gains prominence in later disease stages, particularly in progressive MS phenotypes, when active inflammatory disease activity in terms of contrast-enhancing and active T2 lesions becomes less prominent [3]. The routine evaluation of brain atrophy is clinically meaningful because it has substantial predictive value regarding long-term physical disability, disability progression, and cognitive decline [4,5].

Consequently, neurodegeneration, particularly brain atrophy, is considered an additional treatment target in clinical trials and has been incorporated into the ‘no evidence of disease activity’-4 concept [6]. A change of −0.4% annually has been proposed as the cutoff for pathological brain atrophy in MS [7]. Furthermore, at the group level, accelerated brain volume loss (BVL) early in the disease course positively correlates with the risk of future disability [8]. To date, short-term changes (over 1 year) in brain volume are predictive of clinical status (i.e. diagnosis of MS or disability status) in clinically isolated syndromes (CISs) [9,10], relapsing–remitting MS (RRMS) [11], and primary progressive MS [12], either in isolation or combined with lesion-related parameters [13,14].

In contrast, few studies have evaluated the usefulness of the BVL rate based on the disease duration. These results may provide additional information in the clinical setting, given that the disease duration can be determined prospectively compared with the clinical phenotype, which can be diagnosed retrospectively [15,16].

To date, several studies have evaluated the temporal evolution of BVL in MS. Although the rate of BVL in MS is nearly constant throughout the MS course, regardless of the disease stage, individual differences have been observed [[17], [18], [19], [20]]. Moreover, as the disease duration increases, the risk of chronic progression increases [21], and brain atrophy becomes more evident. Therefore, measuring the prognosis using a classification that considers both BVL rate and disease duration is appropriate.

Therefore, we investigated the relationship between disease duration and annual whole-brain volume changes. Thereafter, we compared the prognoses between groups with higher and lower atrophy rates and similar disease durations. Herein, we evaluated the rate of whole-brain atrophy using ico**brain ms** in the same cohort as that in our previous study [22], which evaluated the regional brain volumes using FreeSurfer analysis. Although our previous study has revealed that baseline volumes of the thalamus and corpus callosum early in the disease stage can predict the progression of brain atrophy in MS, quantification of these regional brain volumes requires complex methods. Therefore, in the current study, we used ico**brain ms**, which can semi-automatically calculate several measurements, to establish a method to predict prognosis in routine clinical practice.

## 2 Materials and methods

### 2.1 Patients

Eighty-two Japanese patients with MS were retrospectively recruited from the Department of Neurology at Tohoku Medical and Pharmaceutical University Hospital, Sendai, Japan, between 2017 and 2021, as previously described [22]. The inclusion criteria were as follows [1]: MS and/or CISs as defined by the 2017 revision of the McDonald criteria [2,23] age between 20 and 70 years. The exclusion criteria were as follows [1]: positivity for anti-aquaporin 4 antibody and/or anti-myelin oligodendrocyte glycoprotein antibody in the serum and/or cerebrospinal fluid determined using a cell-based assay [2], a history of psychiatric illness other than stable depressive symptoms, and [3] a follow-up period of less than half a year. The Expanded Disability Status Scale (EDSS) [24] and Multiple Sclerosis Severity Scale (MSSS) [25] were used to assess the patients’ disability and severity of illness, respectively. In total, 72 of 82 patients with MS agreed to undergo evaluation for information processing speed (IPS) and cognitive assessments using CogEval (Biogen Inc.) (https://apps.apple.com/us/app/cogeval/id1366437045). CogEval is an iPad-based cognitive screening test for MS based on and validated against the Symbol Digit Modalities Test [26,27]. The Institutional Ethics Committee of Tohoku Medical and Pharmaceutical University approved the study protocol (2023-2-002). This study was conducted in accordance with the latest version of the Declaration of Helsinki, which was revised in 2013. All the participants provided informed consent to participate in this study.

### 2.2 Magnetic resonance imaging (MRI) acquisition

The study participants were regularly scanned at least once annually using the same whole-body 1.5-T magnetic resonance imaging (MRI) system (MAGNETOM Aera, Siemens, Germany) with the same MRI protocol. The MR acquisition protocol included a high-resolution sagittal three-dimensional (3D) T1-weighted magnetisation-prepared rapid gradient-echo (MPRAGE) sequence (repetition time [TR], 2730 ms; echo time [TE], 3.3 ms; inversion time [TI], 1000 ms; 176 slices; field of view (FoV), 256 mm; measured isotropic voxel size, 1 × 1 × 1 mm) and a sagittal 3D fluid-attenuated inversion recovery (FLAIR) sequence (TR, 5000 ms; TE, 335 ms; TI, 1800 ms; 176 slices; FoV, 256 mm; and measured isotropic voxel size, 1 × 1 × 1 mm). For assessing brain and lesion volumes at baseline and follow-up, MRI data acquired from patients with a history of relapse or a history of switching or commencing disease-modifying therapies (DMTs) in the past 3 months were excluded from this study (i.e. MRI data from patients who did not meet these exclusion criteria were used).

### 2.3 Measurements of whole-brain, total grey matter, and lesion volumes using a commercially available tool (ico**brain ms**; icometrix, Belgium)

The 3D FLAIR and 3D T1 MPRAGE datasets obtained from each patient were analysed using the program ‘ico**brain ms**’ by uploading the Digital Imaging and Communications in Medicine data to the Icometrix website (http://icometrix.com), as previously described [28]. The ico**brain ms** (Icometrix) is an artificial intelligence software solution for brain MRI analysis in MS [29] and a Conformité Européenne-marked and US Food and Drug Administration-approved proprietary method [8]. The main components of ico**brain ms** are brain tissue and MS lesion segmentation on single-time point T1-weighted and FLAIR scans and specific longitudinal volume change computations for establishing brain atrophy rates and lesion evolution [29]. The ico**brain ms** quantifies cross-sectional volumes with software based on Nifty Seg and quantifies longitudinal changes in the grey and white matter with software that implements Jacobian integration [13]. Compared with other commonly used software, such as FreeSurfer, ico**brain ms** is easier to operate because it semiautomatically calculates brain volumes, although the number of brain regions calculated is smaller. The quality control method includes a quality check of the incoming raw data, a quality check of the resulting segmentation of lesions and brain structures, and the selection of appropriate remarks and consequences for the interpretation of the results. A recent study validated that ico**brain ms** MRI parameters were sensitive to (sub)clinical differences between MS subtypes. Additionally, ico**brain m**s decreased intra- and inter-rater lesion count variability and increased sensitivity in detecting disease activity/progression from 24 to 76% [29]. Using ico**brain ms**, we obtained cross-sectional and longitudinal data on whole-brain volume, total grey matter volume (sum of cortical grey matter volume and deep grey matter volume), T2 lesion load, and T1-hypointense white matter lesion volume. For the longitudinal analysis, we selected MRI images taken at baseline and follow-up. Therefore, the clinical follow-up duration and the duration between the baseline and follow-up MRI scans were similar.

### 2.4 Correlation and cluster analyses

First, we analysed the relationship between the disease duration and whole-brain annualised volume changes by describing the data using a scatter plot. Thereafter, we divided the patients with MS into four groups based on atrophy rate and disease duration. For this purpose, we performed cluster analysis using two variables, whole-brain annualised volume change and disease duration, as optimal cut-offs for the two variables were unavailable. Receiver operating characteristic (ROC) curve analysis was performed to identify the cut-off values for variables that could be used to discriminate between subgroups in which patients with MS would be categorised. To perform comparisons, we identified two groups with higher and lower atrophy rates and similar disease durations.

### 2.5 Statistical analyses

All statistical analyses were performed using JMP Pro version 16.2.0. The Shapiro–Wilk test was used to assess the normality of data distribution. Normally distributed quantitative variables are presented as means (standard deviations), whereas non-normally distributed quantitative variables are presented as medians (interquartile ranges [IQRs]/minimum and maximum). Comparisons of numerical variables between disease groups were performed using the Mann–Whitney *U* test or the Steel–Dwass test, whereas comparisons of categorical variables were performed using the chi-squared tests. Nonparametric correlations between the two quantitative variables were evaluated using Spearman's rank correlation coefficient (rho). To account for type I errors due to multiple comparisons, P values were corrected for false discovery rate (FDR) using the Benjamini–Hochberg method [30]. P values adjusted for the FDR (FDR p values) < 0.05 were considered statistically significant.

## 3 Results

### 3.1 Patient clinical profiles

Eighty-two patients with MS were included in this study. The demographic and clinical data of the study cohort are shown in Table 1. In total, 75 patients with MS (91%) were receiving DMTs at the time of study inclusion.

**Table 1:** Clinical profiles of MS patients.

	Total MS patients (n = 82)	RRMS group (n = 71)	SPMS group (n = 11)	Comparison between RRMS and SPMS groups	
				p value	FDR p value
Disease type during observational period					
CIS→RRMS	n = 1 (1%)	n = 1 (2%)	–		
RRMS→RRMS	n = 65 (79%)	n = 65 (92%)	–		
RRMS→SPMS	n = 5 (6%)	n = 5 (7%)	–		
SPMS→SPMS	n = 11 (14%)	–	n = 11 (100%)		
Sex (F, M)	n = 60, n = 22	n = 53, n = 18	n = 7, n = 4	0.4551	0.579215
Age, mean (SD)^a^	38.9 (8.51)	37.8 (8.06)	45.6 (8.59)	0.00396	**0.009242**
Disease duration, median (IQR)^a^	7.5 (4.2–13.3)	7 [[4], [5], [6], [7], [8], [9], [10], [11]]	20 [[9], [10], [11], [12], [13], [14], [15], [16], [17], [18], [19], [20], [21], [22], [23]]	0.00011	**0.000345**
EDSS, median (IQR)^a^	2 (1–3.5)	2 [[1], [2], [3]]	6 (4.5–7)	3.20E-12	**4.42E-11**
DMT				0.12643	0.22125
Interferon beta (IFNb)	n = 2 (2.5%)	n = 2 (3%)	–		
Dimethyl fumarate (DMF)	n = 21 (26%)	n = 21 (30%)	–		
Fingolimod (FG)	n = 32 (39%)	n = 25 (35%)	n = 7 (64%)		
Natalizumab (NT)	n = 4 (5%)	n = 4 (6%)	–		
IFNb→others (NT (n = 3), DMF (n = 2))	n = 5 (6%)	n = 3 (4%)	n = 2 (18%)		
Glatiramer acetate→others (DMF (n = 2))	n = 2 (2.5%)	n = 2 (3%)	–		
DMF→others (FG (n = 1), NT (n = 2), none (n = 1))	n = 4 (5%)	n = 4 (6%)	–		
FG→others (OFA (n = 1), NT (n = 3), DMF (n = 1)	n = 5 (6%)	n = 4 (6%)	n = 1 (9%)		
None	n = 6 (7%)	n = 5 (7%)	n = 1 (9%)		
None→others (DMF (n = 1))	n = 1 (1%)	n = 1 (1%)	–		
Whole-brain volume (ml), median (IQR)^a^	1504 (1450–1552)	1509 (1471–1560)	1372 (1326–1406)	5.08E-07	**3.56E-06**
Grey matter volume (ml), median (IQR)^a^	884 (857–919)	893 (864–923)	826 (814–864)	0.00012	**0.000345**
T2 lesion load (ml), median (IQR)^a^	4.49 (2.05–10.8)	3.93 (1.67–8.96)	19.51 (8.11–22.8)	0.00004	**0.000183**
Observational period (year), median (IQR)	3.4 (2.3–3.9)	3.33 (2.17–3.83)	3.58 (2.83–3.92)	0.40312	0.564362
ARR during observational period, median (min-max)	0 (0–0.97)	0 (0–0.97)	0 (0–0.63)	0.58022	0.643919
ARR during disease course, median (min-max)	0.23 (0–1.49)	0.22 (0.13–0.37)	0.36 (0.04–1.22)	0.1861	0.289486
Whole-brain annualised volume change (%), median (IQR)	−0.30 (−0.53–−0.20)	−0.3 (−0.53–−0.2)	−0.29 (−0.55–−0.07)	0.59793	0.643919
Grey matter annualised volume change (%), median (IQR)	−0.33 (−0.51–−0.15)	−0.32 (−0.5–−0.17)	−0.43 (−0.58–−0.12)	0.64429	0.644292

a Indicates baseline data. Abbreviations: CIS, clinically isolated syndrome; RRMS, relapsing–remitting MS; SPMS, secondary progressive MS; SD, standard deviation; IQR, interquartile range; EDSS, Expanded Disability Status Scale; DMT, disease-modifying therapy; ARR, annualised relapse rate; FDR p value, p value adjusted for the false discovery rate.

The median observational period was 3.4 (IQR, 2.3–3.9) years. During the follow-up period, one patient with CISs converted to RRMS and five patients converted from RRMS to SPMS, based on the definition provided by Lublin et al. [31]. We divided the MS patient group (n = 82) into two subgroups [1]: the RRMS (CISs to RRMS, n = 1; RRMS to RRMS, n = 65; RRMS to SPMS, n = 5) and SPMS (SPMS to SPMS, n = 11) groups.

Patients in the SPMS group were significantly older and had a longer disease duration and higher EDSS scores than those in the RRMS group. In contrast, there were no differences in the distribution of sex, DMT, observational period, or annualised relapse rate (ARR) during the observational period or disease course between the RRMS and SPMS groups.

### 3.2 MRI measurements

The median whole-brain and grey matter annualised volume changes of all patients during the observational period were −0.30 (IQR, −0.53 to −0.20) and −0.33 (−0.51 to −0.15), respectively (Table 1). Patients in the SPMS group had significantly lower whole-brain and grey matter volumes and higher T2 lesion loads than patients in the RRMS group. In contrast, whole-brain and grey matter annualised volume changes did not differ significantly between the RRMS and SPMS groups.

### 3.3 Correlation and cluster analyses

**Fig. 1:**
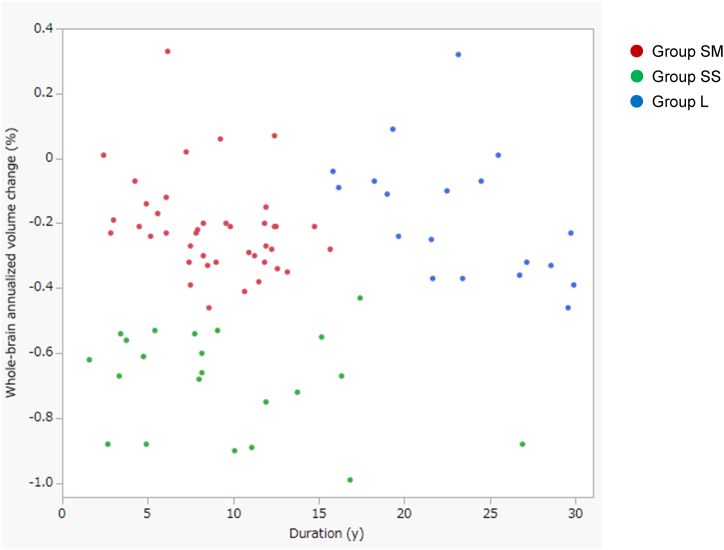
Scatterplot illustrating the distribution of the three groups. The scatterplot shows that the three groups (SM, SS, and L) are separated from each other. The SM group included patients with a short to middle duration and mild atrophy rates, the SS group included patients with a short to middle duration and severe atrophy rates, and the L group included patients with a long duration.

The scatterplot shows that the whole-brain annualised volume change significantly differed among patients with a short to middle disease duration (Fig. 1).

The smooth curve fitted using the locally estimated scatterplot smoothing regression technique indicated that the annualised volume changes in the whole brain remained constant regardless of disease duration (data not shown). The annualised volume change in the whole brain did not correlate with disease duration (rho = 0.075).

Cluster analysis using two variables, whole-brain annualised volume change and disease duration, showed that patients with MS could be classified into three groups, although we expected them to be divided into four groups (eFig. 1). The scatterplot shows that the three groups were divided based on disease duration and annualised BVL rate (Fig. 1). Therefore, we defined the three groups as follows: SM (short to middle duration and mild atrophy rates), SS (short to middle duration and severe atrophy rates), and L (long duration) groups. Regarding the whole-brain annualised volume change, ROC curve analysis revealed an optimal cut-off value of −0.43, which discriminated the SS group from the SM and L groups, resulting in 100% sensitivity and 97% specificity. For disease duration, ROC curve analysis revealed an optimal cut-off value of 15.8, which discriminated the L group from the SM and SS groups, resulting in 100% sensitivity and 94% specificity (Table 2).

**Table 2:** Cut-off values for distinguishing group SS or group L from others.

	Cut-off value	AUC	Sensitivity	Specificity
SS vs. others (SM and L)	whole-brain annualised volume change = −0.43 (%)	0.998	1	0.97
L vs. others (SM and SS)	disease duration = 15.8 (y)	0.983	1	0.94

SM: short to middle duration and mild atrophy rates; SS: short to middle duration and severe atrophy rates; L: long duration; AUC: area under the curve.

### 3.4 Comparison of the SM, SS, and L groups

**Fig. 2:**
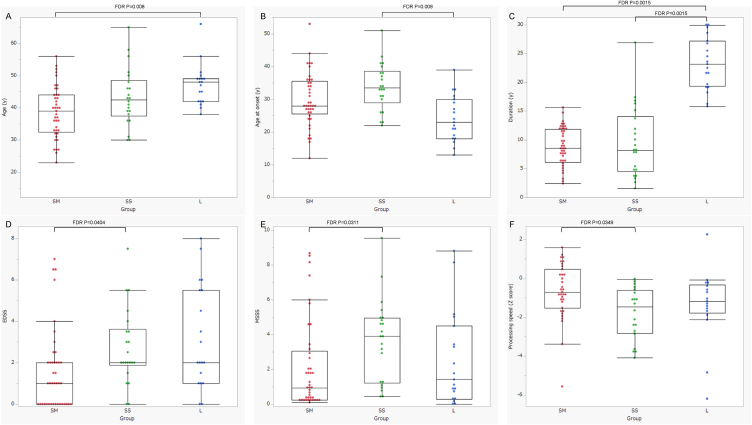

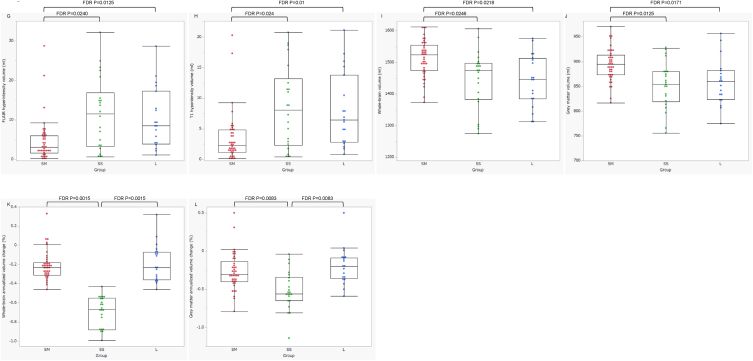
Comparison of clinical and radiological findings among the three groups. Compared with patients in the other two groups, those in the SM group show lower lesion loads (G), T1-hypointense white matter volumes (H), and whole-brain and grey matter volume loss (I, J). The EDSS and MSSS scores are significantly lower in the SM group than in the SS group (D, E). The IPS, evaluated in 72 patients with MS, is significantly lower in the SS group than in the SM group (F). In contrast, patients in the SS group have significantly higher annualised changes in whole-brain and grey matter volumes than those in the other two groups (K, L). Patients in the group L exhibit a longer disease duration than those in the other two groups (C). In addition, patients in the L group are significantly older than those the SM group (A), whereas age at disease onset is significantly lower in the L group than in the SS group (B). FDR, false discovery rate.

Detailed comparisons of the three groups are presented in Table 3. Compared with the other two groups, the SM group had lower lesion loads (Fig. 2G), T1-hypointense white matter volume (Fig. 2H), and whole-brain and grey matter volume loss (Fig. 2I and J). The EDSS and MSSS scores were significantly lower in the SM group than in the SS group (Fig. 2D and E). Although the IPS was evaluated in 72 patients with MS, it was significantly lower in the SS group than in the SM group (Fig. 2F), whereas age and educational level did not differ significantly between the two groups (Table 3). In contrast, the SS group had significantly higher annualised volume changes in the whole brain and grey matter than the other two groups (Fig. 2K and L). The L group had a longer disease duration than the other two groups (Fig. 2C). In addition, age was significantly higher in the L group than in the SM group (Fig. 2A), whereas the age at disease onset was significantly lower in the L group than in the SS group (Fig. 2B).

**Table 3:** Comparison of clinical and radiological findings among the 3 groups at follow-up.

				p values			FDR-corrected p values		
	Group SM (n = 41)	Group SS (n = 22)	Group L (n = 19)	L vs. SM	SS vs. SM	L vs. SS	L vs. SM	SS vs. SM	L vs. SS
Age (y)	39 (32.5–44)	42.5 (37.5–48.5)	48 (42–49)	**0.0008**	0.1958	0.1761	**0.0080**	0.3455	0.3202
Age at onset (y)	28 (25.5–35.5)	33.5 (29–38.5)	23 [[18], [19], [20], [21], [22], [23], [24], [25], [26], [27], [28], [29], [30]]	**0.032**	0.1633	**0.0007**	0.0711	0.3062	**0.0080**
Sex (F, M)	29 (71%), 12 (29%)	16 (73%), 6 (27%)	15 (79%), 4 (21%)		0.7988			0.9976	
Duration (y)	8.6 (6.1–11.9)	8.2 (4.5–14.1)	23.2 (19.3–27.2)	**<0.0001**	0.9927	**<0.0001**	**0.0015**	0.9976	**0.0015**
EDSS	1 (0–2)	2 (1.9–3.6)	2 (1–5.5)	**0.0252**	**0.0155**	0.9272	0.0582	**0.0404**	0.9976
MSSS	0.94 (0.25–3.05)	3.9 (1.22–4.94)	1.42 (0.28–4.49)	0.8307	**0.0109**	0.2053	0.9976	**0.0311**	0.3519
ARR (observation period)	0 (0–0)	0 (0–0.34)	0 (0–0)	0.0756	0.5356	**0.0231**	0.1463	0.7987	0.0554
ARR (disease course)	0.22 (0.13–0.33)	0.25 (0.13–0.50)	0.26 (0.11–0.47)	0.6816	0.4635	0.9428	0.9187	0.7131	0.9976
Disease course					0.0569			0.1219	
CIS→RRMS	n = 1 (2.5%)	n = 0 (0%)	n = 0 (0%)						
RRMS→RRMS	n = 37 (90%)	n = 16 (72%)	n = 12 (63%)						
RRMS→SPMS	n = 1 (2.5%)	n = 3 (14%)	n = 1 (5%)						
SPMS→SPMS	n = 2 (5%)	n = 3 (14%)	n = 6 (32%)						
DMTs					0.689			0.9187	
High-efficacy	n = 17 (42%)	n = 8 (36.5%)	n = 11 (58%)						
Platform	n = 10 (24%)	n = 8 (36.5%)	n = 5 (26%)						
None	n = 3 (7%)	n = 2 (9%)	n = 1 (5%)						
Switch	n = 11 (27%)	n = 4 (18%)	n = 2 (11%)						
FLAIR hyperintensity vol*	3.13 (1.67–6.06)	11.54 (3.29–16.87)	8.58 (3.9–17.27)	**0.0026**	**0.0076**	0.9924	**0.0125**	**0.0240**	0.9976
T1 WM hypointensity vol*	2.33 (1.16–4.85)	8.09 (2.32–13.23)	6.44 (2.85–13.8)	**0.0015**	**0.0075**	0.9954	**0.0100**	**0.0240**	0.9976
Whole-brain vol*	1524 (1474–1555)	1475 (1383–1498)	1447 (1385–1513)	**0.0058**	**0.0082**	0.9954	**0.0218**	**0.0246**	0.9976
Grey matter vol*	895 (874–914)	855 (819–881)	860 (824–882)	**0.004**	**0.0025**	0.9472	**0.0171**	**0.0125**	0.9976
Whole-brain annualised vol change (%)	−0.23 (−0.31–−0.18)	−0.67 (−0.88–0.55)	−0.23 (−0.36–−0.07)	0.9319	**<0.0001**	**<0.0001**	0.9976	**0.0015**	**0.0015**
Grey matter annualised vol change (%)	−0.31 (−0.4–−0.14)	−0.56 (−0.65–−0.34)	−0.2 (−0.36–−0.09)	0.5458	**0.001**	**0.0011**	0.7987	**0.0083**	**0.0083**

				p values			FDR-corrected p values		
	Group SM (n = 35)	Group SS (n = 20)	Group L (n = 17)	L vs. SM	SS vs. SM	L vs. SS	L vs. SM	SS vs. SM	L vs. SS
Age (y)	40 (33–46)	42.5 (38.3–49.5)	48 (42–50.5)	**0.0052**	0.2407	0.2925	**0.0208**	0.4012	0.4743
Education (y)	14 [[12], [13], [14], [15]]	13.5 [[12], [13], [14], [15], [16]]	12 [[12], [13], [14]]	0.7076	0.9197	0.6007	0.9230	0.9976	0.8382
IPS (raw score)	62 (50–67)	48 (41–59)	51 (47–58)	0.0702	**0.0069**	0.8391	0.1452	**0.0240**	0.9976
IPS (Z score)	−0.72 (−1.52-0.47)	−1.48 (−2.83–0.613)	−1.18 (−1.78–0.33)	0.3284	**0.0128**	0.5925	0.5185	**0.0349**	0.8382

SM: short to middle duration and mild atrophy rates; SS: short to middle duration and severe atrophy rates; L: long duration; WM: white matter; vol: volume; IPS: information processing speed. Asterisks indicate that the units are ml. Bold indicates p values less than 0.05.

### 3.5 Correlation between whole-brain annualised volume change and clinical scores in 63 patients in the SM and SS groups

**Fig. 3:**
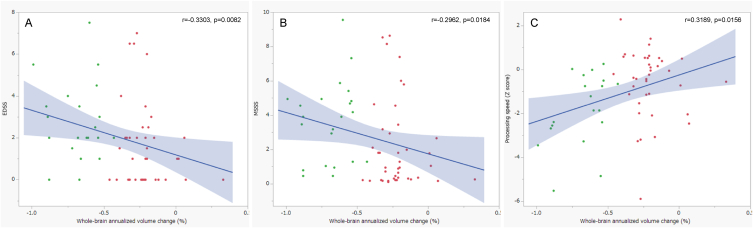
Correlation between whole-brain annualised volume change and clinical scores in 63 patients in the SM and SS groups. Although the EDSS, MSSS, and IPS z-scores do not significantly correlate with the whole-brain annualised volume changes in our full set of 82 patients with MS, they significantly correlate upon excluding patients in the L group.

Although the EDSS, MSSS, and IPS z-scores did not significantly correlate with the whole-brain annualised volume change in 82 patients with MS, they significantly correlated upon excluding patients in the L group (Fig. 3).

## 4 Discussion

To the best of our knowledge, this is the first study to investigate the rate of BVL in routine clinical practice using ico**brain ms** in a relatively large group of Japanese patients with MS. We used an automated web-based platform, ico**brain ms**, capable of quantifying longitudinal BVL at high levels of statistical agreement and consistency using the Structural Image Evaluation using Normalisation of Atrophy (SIENA) package in this real-world MS population [8]. SIENA has been and will be extensively utilised in longitudinal MS studies, although its implementation in routine clinical practice is limited by the need for manual image preprocessing by trained image analysts and the lack of a user interface accessible to nonexperts [8].

In our previous study, we utilised a hierarchical cluster analysis with multivariate imaging data obtained by FreeSurfer analysis and classified cross-sectionally patients with MS into three clusters (Clusters 1, 2, and 3) in ascending order by disability and BVL at baseline [28] and follow-up [22]. Among patients included in Cluster 1 at baseline, approximately one-third of patients transitioned into Cluster 2 at follow-up. Baseline volumes of the corpus callosum, thalamus, and whole brain, excluding the volume of the ventricles, were significantly reduced in the transition group when compared with those in the nontransition group and were identified as the most important predictors of transition [22]. Conversely, in the current study, we found that the rate of whole-brain atrophy could be easily obtained in routine clinical practice using icobrain ms, which could be another good prognostic predictor.

Herein, we observed that whole-brain annualised volume changes varied, especially among patients with short to middle disease duration. However, the whole-brain annualised volume change did not statistically correlate with disease duration and remained relatively constant regardless of disease duration, as reported in previous studies [17]. We also found that whole-brain and grey matter annualised volume changes did not differ significantly between patients with RRMS and SPMS, as suggested in previous studies [19].

Thereafter, we compared the two groups with higher and lower atrophy rates and similar disease durations. For this purpose, we performed cluster analysis and found that patients with MS in our cohort could be classified into three groups by applying the following optimal cut-off values: a disease duration of 15.8 years and an annualised whole-brain volume change of −0.43%. Patients with MS with a disease duration of <15.8 years were classified into two groups, defined by high and low whole-brain atrophy rates. Compared with patients in the SM group, patients in the SS group had significantly higher MSSS and EDSS scores, lower IPS, higher lesion loads and T1-hypointense white matter volumes, and higher volume loss and atrophy rates in the whole brain and grey matter. The proportion of patients who converted from RRMS to SPMS was slightly higher in the SS group than in the control group. Moreover, among the 63 patients with MS who were included in the SM and SS groups, whole-brain annualised volume changes were significantly correlated with the EDSS and MSSS scores and IPS. These results suggest that the rate of brain atrophy, as evaluated in routine clinical practice using ico**brain ms**, can be used to assess patient prognosis.

To date, several predictors of poor prognosis in MS have been suggested, and high-efficacy therapies are recommended for individuals with poor prognostic features. The predictors include demographic, environmental, and clinical factors, MRI observations, and biomarkers associated with a poor prognosis in MS [32]. In our study, among the suggested predictors of poor prognosis, patients in the SS group had larger T2 lesion volumes, higher rates of whole-brain and grey matter atrophy, higher EDSS scores, and greater early cognitive deficits than those in the SM group. Moreover, although not statistically significant, patients in the SS group were relatively older at onset and had a relatively higher ARR than those in the SM group. In the future, we need to evaluate the characteristics of the patients in the SS group in detail, such as comorbidities, lifestyle factors, and the distribution of lesions in the brain and spinal cord.

Our study has some limitations. First, this was a single-centre study conducted in a limited sample of Japanese patients with MS with a short follow-up duration; therefore, the results may have been influenced by selection bias. Furthermore, this categorisation scheme may not be appropriate for other cohorts, as Japanese patients with MS present with a milder clinical course [33] and lower proportions of secondary progressive MS [[34], [35], [36]] and primary progressive MS [33,35,36] than Caucasian patients. Second, our study mostly included patients receiving DMTs; therefore, the effects of pseudoatrophy cannot be completely avoided. Moreover, our study included a relatively high proportion of patients taking fingolimod and dimethyl fumarate, which may have affected the generalisability of the results. Third, our cohort did not include patients with MS with both a longer disease duration and severe brain atrophy rate, whereas these patients were observed in previous Caucasian studies [17,20]. Although we attributed these results to ethnic differences, we speculated that these patients could not be included because they tended to have reached nursing homes or needed home care owing to poor activities of daily living and eventually could not regularly visit the hospital. Further multicentre analyses, including a large number of patients with MS, are required. Fourth, because the clinical follow-up duration and the duration between successive MRI scans were similar in our study, we evaluated the prognosis immediately after the follow-up MRI. Because we need to predict the prognosis several months after follow-up MRI in the clinical setting, additional studies are required.

## 5 Conclusions

Whole-brain annualised volume changes vary, especially among Japanese patients with MS with a disease duration of approximately 16 years. Among them, patients with an increased rate of brain atrophy show increased physical and cognitive impairments, along with MRI findings suggesting active neuroinflammation and neurodegeneration.

## Declarations

### Ethical statement

The Institutional Ethics Committee of Tohoku Medical and Pharmaceutical University approved the study protocol (2023-2-002). All the participants provided informed consent to participate in this study.

## Data availability statement

Individual-level data are not publicly available because of legal restrictions. However, some anonymised data supporting the findings of this study are available upon request from the corresponding author.

## CRediT authorship contribution statement

**Juichi Fujimori:** Writing – review & editing, Writing – original draft, Visualization, Project administration, Methodology, Investigation, Formal analysis, Data curation, Conceptualization. **Ichiro Nakashima:** Writing – review & editing, Validation, Supervision, Resources, Funding acquisition.

## Declaration of competing interest

IN serves on scientific advisory boards for Biogen Japan and Novartis Pharma and receives honoraria for speaking engagements with Biogen Japan, Mitsubishi Tanabe Pharma, Novartis Pharma, Takeda Pharmaceutical, and Eisai. JF declares no conflicts of interest.
