# Supplementary material for: Brain volume loss in Japanese patients with multiple sclerosis is present in the early to middle stage of the disease
Source: Heliyon. 2024 Mar 18;10(6):e28136. doi: 10.1016/j.heliyon.2024.e28136 (PMC10965524; doi:10.1016/j.heliyon.2024.e28136)
Supplement: Multimedia component 1 [file mmc1.docx]

eFigure 1


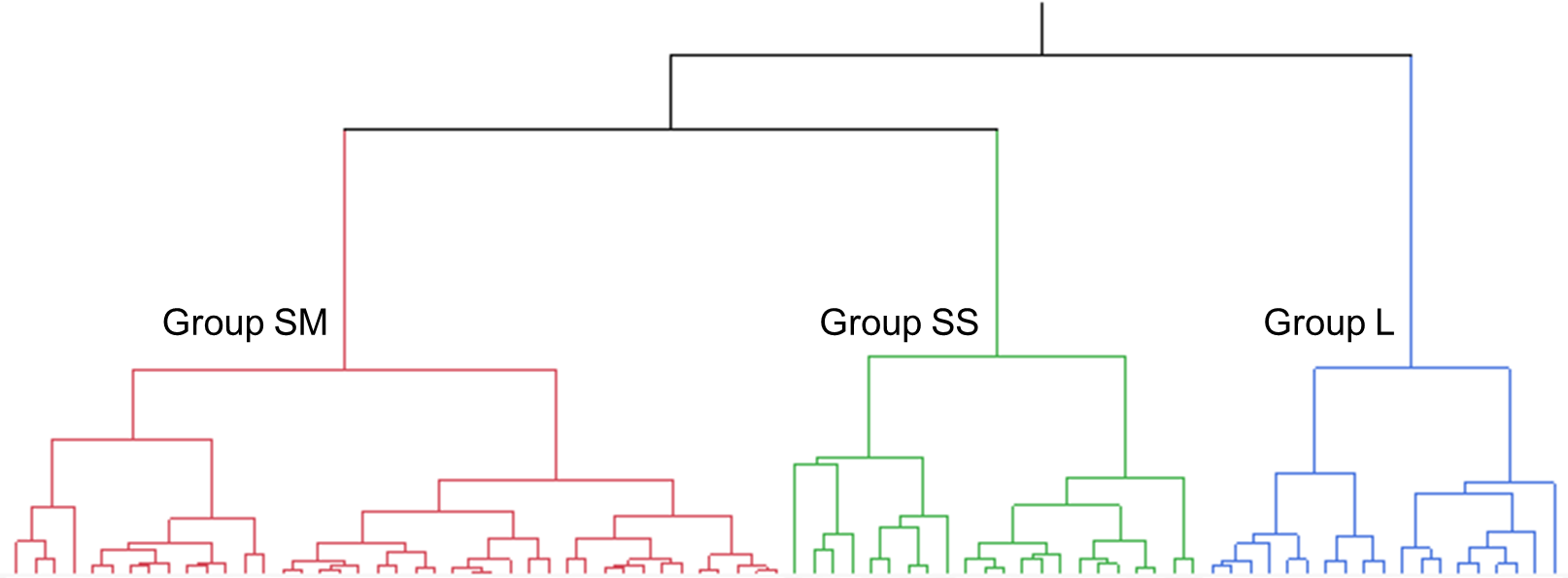


eFigure 1. Cluster analysis of 82 MS cases using two variables (whole-brain annualized volume change and disease duration) revealed that MS was classified into three groups (Groups SM, SS, and L). Group SM: patients with a short to middle duration and mild atrophy rates; Group SS: patients with a short to middle duration and severe atrophy rates; Group L: patients with a long duration.
